# Effects of Pulsed Electric Field and High-Pressure Processing Treatments on the Juice Yield and Quality of Sea Buckthorn

**DOI:** 10.3390/foods13121829

**Published:** 2024-06-11

**Authors:** Zhiwei Zhang, Yixuan Chen, Yuying Cheng, Zhenhong Gao, Kunsheng Qu, Zhixi Chen, Lihua Yue, Wenqiang Guan

**Affiliations:** 1 Tianjin Key Laboratory of Food Biotechnology, School of Biotechnology and Food Science, Tianjin University of Commerce, Tianjin 300134, China; zzw5301@163.com (Z.Z.); cyx1061607620@163.com (Y.C.); 15866561201@163.com (Y.C.); 2 School of Biotechnology and Food Science, Tianjin University of Commerce, Tianjin 300134, China; gzh9290@163.com (Z.G.); qukunsheng@126.com (K.Q.); 3 Huachi Gannong Biotechnology Company Limited, Qingyang 745600, China; 18919962872@163.com; 4 Chengde Astronaut Mountainous Plant Technology Company Limited, Chengde 068450, China; yuelihua9999@163.com

**Keywords:** high-pressure processing (HPP), juice yield, pulsed electric field (PEF), sea buckthorn juice, volatile organic compounds (VOCs)

## Abstract

Sea buckthorn juice has high nutritional value and a rich flavor that consumers enjoy. Traditional sea buckthorn thermal processing (TP) technology has problems such as low juice yield, poor quality, and poor flavor. Sea buckthorn berries are processed using a technique combining pulsed electric field (PEF) and high-pressure processing (HPP) to increase juice yield and study its impact on the quality and volatile aroma of sea buckthorn juice. Results have show that, compared with TP, under the condition of PEF-HPP, the juice yield of sea buckthorn significantly increased by 11.37% (*p* > 0.05); TP and PEF-HPP treatments could effectively kill microorganisms in sea buckthorn juice, but the quality of sea buckthorn juice decreased significantly after TP treatment (*p* > 0.05), whereas PEF-HPP coupling technology could maximally retain the nutrients of sea buckthorn juice while inhibiting enzymatic browning to improve color, viscosity, and particle size. The flavor of sea buckthorn juice is analyzed using electronic nose (E-nose) and gas chromatography–ion mobility spectrometer (GC–IMS) techniques, and it has been shown that PEF-HPP retains more characteristic volatile organic compounds (VOCs) of sea buckthorn while avoiding the acrid and pungent flavors produced by TP, such as benzaldehyde, (E)-2-heptenal, and pentanoic acid, among others, which improves the sensory quality of sea buckthorn juice. PEF-HPP technology is environmentally friendly and efficient, with significant economic benefits. Research data provide information and a theoretical basis for the sea buckthorn juice processing industry.

## 1. Introduction

Sea buckthorn (*Hippophae rhamnoides* L. or *Elaeagnus rhamnoides* L.), also known as vinegar willow, sour thorn, dapple, and so on, is a deciduous shrub or tree in the family Hippophae, belonging to small berry fruit trees [1]. Sea buckthorn is native to cold-temperature regions of Asia and Europe, although it is currently primarily dispersed in 45 nations and regions with moderate climates in Eurasia [2]. As a medicinal and food plant, the whole plant tissue of sea buckthorn contains approximately 200 active components, including various types of vitamins, flavonoids, phenolic compounds, amino acids, fatty acids, and tannins [3]. Sea buckthorn contains the most polyphenols, especially phenolic acids and flavonoids, and the amount of vitamins and flavonoids is several times that of oranges, hawthorns, and blueberries. Sea buckthorn has been used in medical treatment due to its high fatty acid content, which improves cardiovascular health and raises cholesterol [4]. Sea buckthorn juice can retain its original nutritional value, and it is convenient to drink. These properties make it dominate among sea buckthorn drinks. However, as freshly squeezed sea buckthorn juice has a short shelf life, some processing processes must be utilized to extend it. Sterilization is an essential step in the processing of fruit and vegetable juice, as it directly determines the quality and safety of the juice [5].

Food processing technology is broadly classified into two types: thermal and non-thermal processing. The most typical way is to sterilize the juice through thermal processing (TP), but TP reduces the juice’s natural nutritional value, appearance quality, and scent flavor owing to the high temperature [6,7]. To solve this issue, non-thermal processing technology substitutes for TP have been identified to maximize consumer demand for excellent nutritional quality and an appealing flavor and look.

Pulsed electric field (PEF) is a novel processing technology that applies electrical energy in the form of high-intensity, very short pulses to food products using a specially designed voltage-producing power generator [8]. It has been reported that 0.5 kV can cause irreversible electroporation of cell membranes at critical field strengths and increase membrane permeability and diffusion of intracellular molecules [9]. PEF (1–6 kV/cm) synergizes with mechanically pressed raspberries to increase juice yield by 9–25% [10]. Synergistic solid–liquid extraction with PEF (1–10 kJ/kg, 3 kV/cm) for processing blueberry byproducts is adequate to dramatically boost blueberry juice yield by 32% [11]. Li et al. used PEF (30 kV/cm, 400 μs) for sterilization in cantaloupe juice, and Rian et al. used PEF (0.9 kV/cm and 2.7 kV/cm) as an alternative to TP for processing orange juice. Both were found to prolong the shelf-life of the juices, maintain the physicochemical properties of the food products, and retain the nutrients and freshness of the flavor [12,13]. PEF has attracted increasing attention in food processing for its ability to preserve heat-sensitive nutrients and volatile organic compounds (VOCs), maximizing the retention of color, aroma, VOCs, and nutrients in food.

High-pressure processing (HPP) involves using high pressure in a closed environment, as a result of which the microorganisms in food cannot perform their cellular functions properly, leading to protein denaturation, enzyme inactivation, and cellular membrane breakage [14]. High-pressure conditions distort cellular structures and change cell membrane permeability, resulting in cell death [15]. HPP treatment of carrot juice (600 MPa, 3 min, 20 °C) and persimmon juice (300 MPa, 8 min) reduced enzymatic browning of fruits and vegetables while inactivating microorganisms and maintaining nutrients, including total phenols and antifolate [16,17]. HPP can be used instead of TP to improve the shelf-life of apple and orange juices while still providing consumers with fresh sensory attributes [18].

Sea buckthorn juice, being liquid and not an insulator, faces challenges during PEF processing, as high electric field strengths can lead to abnormal breakdown of the treated foodstuffs and contamination of the liquid foodstuffs [19]. Endogenous enzymes that are resistant to compression, such as polyphenol oxidase (PPO) and pectin methylesterase, can only be slightly inhibited under HPP conditions [20]. There have been few studies on the relevance of HPP’s direct effect on juice to improve juice yield. Therefore, it is possible to boost juice yield by shattering the cells with PEF technology and employing HPP processing to ensure food safety. To the best of our knowledge, combining PEF and HPP to produce sea buckthorn juice has not been reported.

We evaluated the effects of PFE-HPP and TP on the quality characteristics of sea buckthorn juice, and the changes of VOCs in sea buckthorn juice were monitored using the headspace gas chromatography–ion mobility spectrometer (HS-GC–IMS) technique. This work’s purpose was to develop PEF-HPP synergistic technology as a new non-thermal processing method to solve the problem of a low yield of sea buckthorn juice, improve its appearance and nutritional quality, and maintain good flavor, which will provide new thoughts for the development of the sea buckthorn industry.

## 2. Materials and Methods

### 2.1. Raw Material

Fresh sea buckthorn berries were obtained from Chinese sea buckthorn in Huachi County, Gansu Province, China. Randomly selected sea buckthorn berries with uniform color, moderate size, and maturity were examined. These berries were stored at −26 °C in 500 g bags and then thawed under running water before the testing to wash away the soil and impurities.

### 2.2. Preparation of Sea Buckthorn Juice and Treatment

During the pre-test period, PEF and HPP treatment parameters were optimized. The results showed no microbial colonies in the total bacterial count test and the highest juice yield under the conditions of PEF at 5000 V, frequency of 50 Hz, HPP at 500 MPa, and 20 °C for 15 min.

**Figure 1:**
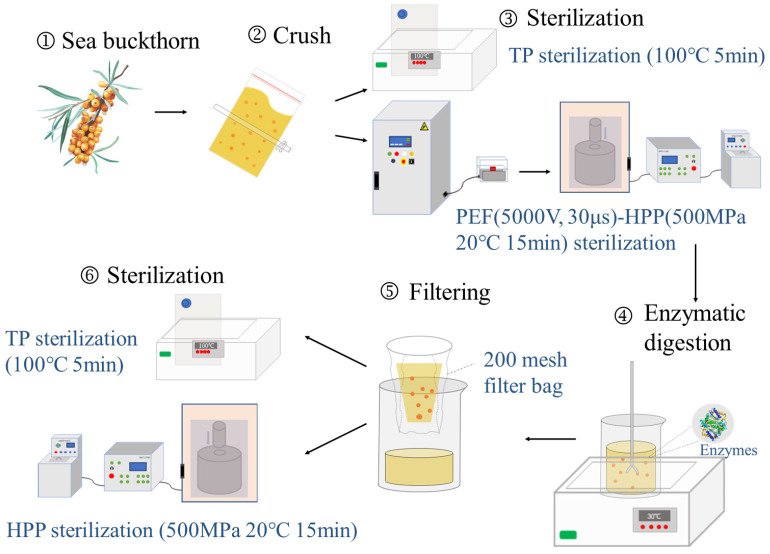
Flow chart depicting the production of sea buckthorn juice.

The sea buckthorn berries were crushed to extract the juice and then sterilized. The sterilized sea buckthorn juice and pomace underwent enzymatic digestion (pectinase and cellulase) in a water bath at 30 °C for 1 h, followed by filtration to obtain the sea buckthorn juice, which was again sterilized (Figure 1). Three samples of sea buckthorn juice were prepared: control check (CK): without any treatment; TP: sterilization in a boiling water bath at 100 °C for 5 min; PEF-HPP: PEF treatment with a pulse voltage of 5000 V, frequency of 50 Hz, spacing of 5 cm between the two electrode plates, time 30 μs, followed by HPP (Huatai Senmiao Bioengineering Technology Co., Tianjin, China) treatment at 500 MPa, 20 °C, and a holding pressure for 15 min.

### 2.3. Determination of Aerobic Plate Count

The full-plate count method was used to determine the aerobic plate count in sea buckthorn juice. The experimental method and procedure were based on the Chinese Standard GB/T 4789.2-2022 [21]. Three gradient dilutions were prepared by diluting the sea buckthorn juice sample with sterilized saline. Afterward, 1 mL of the dilution was pipetted and poured into 15 mL of plate count agar, shaken well, and allowed to set before inverting, and finally cultured at 37 °C for 48 h. Viable counts were expressed as CFU/mL. The counting formula of aerobic plate count was as follows:

(1) $$N=\frac{\sum C}{{(n}_{1}+0.1n_{2})\times d}$$

where *N* is the aerobic plate count in the sample, *∑C* is the sum of the number of aerobic plate count on the plates, *n*_1_ is the number of plates at the first dilution, *n*_2_ is the number of plates at the second dilution, and *d* is the dilution factor.

### 2.4. Juice Yield

Sea buckthorn fruits weighing 500 g were used. After removing any rotten fruit and debris, the weight was recorded as *M*_1_. The weight of the pulp after the second sterilization was recorded as *M*_2_. The juicing rate of sea buckthorn was then calculated using the following equation [10]:

(2) $$\mathrm{Juicing}\;\mathrm{rate}=\frac{M_{1}}{M_{2}}\times 100\%$$

### 2.5. Nutritional Quality Analysis

#### 2.5.1. Total Soluble Solid Content and Titratable Acidity

Total soluble solid (TSS) content and titratable acidity (TA) were determined at 25 °C ± 1 °C using a PAL-BX/ACID1 sugar–acid integrated machine (ATAGO, Co., Ltd., Tokyo, Japan) [22]. TSS results were expressed as Brix, and TA results were expressed as g/100 g based on citric acid.

#### 2.5.2. Determination of Ascorbic Acid

The ascorbic acid content in sea buckthorn juice was determined using the 2, 6-dichlorophenolindophenol assay (Chinese Standard GB 5009.86-2016) [23]. Extraction of ascorbic acid was carried out using 5 mL of 5 g/100 g trichloroacetic acid solution and 1 mL of sea buckthorn juice. After centrifugation, the supernatant was filtered and titrated with 0.1 g/100 g aqueous sodium dichlorophenolindophenol solution.

#### 2.5.3. Determination of Total Phenolics

The total phenol content of sea buckthorn juice was determined using the Folin–Ciocalteu method [24]. In total, 5 mL of sea buckthorn juice was mixed with 20 mL of an 80% methanol solution, subjected to 15 min of ultrasonication, and then centrifuged at 10,000 rpm and 4 °C for 15 min. After a 10-fold dilution of the supernatant, 0.4 mL of the extract were combined with 2 mL of a 10-fold diluted forintanol reagent and 1.8 mL of a 7.5% Na_2_CO_3_ solution. The reaction was carried out at room temperature for 1 h, and the absorbance was measured at 765 nm. The total phenol content was expressed as a milligram gallic acid equivalent per 100 g of the sample.

#### 2.5.4. Determination of Total Flavonoids

The AlCl_3_-NaNO_2_ colorimetric method was utilized to ascertain the total flavonoid concentration in sea buckthorn juice [25]. After 10-fold dilution of sea buckthorn juice, 1 mL of the sample solution was taken, added to 0.3 mL of 5% NaNO_2_ and 10% AlCl_3_ successively, and then left to sit for 6 min each. To determine the absorbance value at 510 nm, 4 mL of 10% NaOH were added, the sample was fixed to 10 mL with distilled water, and it was then left for 15 min. Rutin was used as the standard sample for calculating the total flavonoid content.

#### 2.5.5. Analysis of Organic Acid Content

The organic acid content was determined by the methods outlined in Chinese Standard GB 5009.157-2016 [26]. Sea buckthorn juice samples, each weighing 5 g, were filtered through a 0.45 μm filter membrane. High-performance liquid chromatography (HPLC) with a photodiode array detector (HPLC-PDA; Waters Corporation, Milford, MA, USA) was used for the analysis. The chromatographic column was a C18 column (4.6 × 250 mm^2^, 5 μm, ZORBAX Eclipse XDB, Agilent, Santa Clara, CA, USA), and the mobile phase was 0.1 mol/L H3PO4-methanol (97.5: 2.5, *v*/*v*) at a flow rate of 1.0 mL/min with the column temperature maintained at 40 °C. The injection volume was 10 μL, the detection wavelength was 210 nm, and the detection time was 15 min.

### 2.6. Analysis of Color Characteristics

The chromaticity values, including L* (brightness), a* (red–green), and b* (yellow–blue), were determined using a colorimeter (CR-400, Konica Minolta, Tokyo, Japan). Before taking measurements, the colorimeter was calibrated using a white reference tile at ambient temperature. The total color difference (ΔE*), chroma (C*), and hue angle (*h*^0^) between the untreated sea buckthorn juice and the treated samples was calculated using the following Equations (3)–(5) [25]:

(3) $$\Delta E*=\sqrt{{{\left(\Delta L*\right)}^{2}+\left(\Delta a*\right)}^{2}+{\left(\Delta b*\right)}^{2}}$$

(4) $$C*=\sqrt{{\left(a*\right)}^{2}+{\left(b*\right)}^{2}}$$

(5) $$h^{0}=\arctan\frac{b*}{a*}$$

### 2.7. Particle Size Distribution

The particle size distribution was determined using a Mastersizer 3000 laser particle sizer (Malvern Instruments Limited, Malvern, UK) [27]. The shading rate was 5–15%. The particle type was spherical. The sample’s refractive index was 1.471, its density was 1.03 g/mL, and the refractive index of the dispersant (water) was 1.33. The distribution of the mean diameter of the particle sizes D[3,2] and D[4,3] was analyzed.

### 2.8. Determination of Viscosity

The viscosity of sea buckthorn juice was measured using an NDJ-5S rotary digital viscometer (Shanghai Xinniu Instrument Co., Ltd., Shanghai, China), and the findings were given in mPa·S. The viscosity of sea buckthorn juice was measured with a rotor No. 0 at a room temperature of 25 °C for 1 h. Sea buckthorn juice was poured into a sample cup at room temperature 25 °C for 1 h and then measured using rotor 0. The surface tension angle was regulated in the region of 45–50% [28].

### 2.9. Enzyme Activities

Peroxidase (POD) and PPO relative activities were determined using the methods proposed by Kalsi et al. [29] and Koo et al. [30]. The samples of sea buckthorn juice were centrifuged at 12,000 rpm and 4 °C for 20 min for the enzyme activity assay. To assess the POD activity, we prepared a mixture that included 0.1 mL of crude enzyme extract, 2.15 mL of 0.5% guaiacol solution (pH = 6.0), and 0.25 mL of H_2_O_2_ (0.1%). The increase in absorbance at 470 nm within 3 min was then recorded. To determine the PPO activity, 0.3 mL of crude enzyme extract were mixed with 2 mL of buffer (containing 1 mmol polyethylene glycol, 4% polyvinylpyrrolidone, and 1% Triton X-100 and 0.7 mL catechol (50 mmol/L). The absorbance was recorded at 400 nm within 3 min. The relative residual activity was calculated using the following formula: relative residual activity = (activity of treated samples/activity of untreated sea buckthorn juice) × 100%. The superoxide dismutase (SOD) activity was determined using the Chinese Standard GB/T 41906-2022 for the o-phenyltriol autoxidation method, and the results were represented as one SOD activity unit (U) [31].

### 2.10. Determination of Volatile Compounds

#### 2.10.1. Determination of Volatile Compounds Using E-Nose

A 2 mL sample of sea buckthorn juice was placed in a 20 mL headspace vial and incubated at 60 °C for 20 min. The injection volume was 2000 μL, the injection rate was 125 μL/s, the temperature of the injection port was 200 °C, the outlet flow rate was 30 mL/min, the injection time was 45 s, the cleaning time was 200 s, and the data acquisition time was 110 s [32]. The compounds were calibrated using n-alkane standard liquid, and the data were processed using Alpha Soft Version 14.5.

#### 2.10.2. Determination of the Content of Volatile Compounds Using HS-GC–IMS

The GC–IMS analysis was carried out by a FlavourSpec GC–IMS system (G.A.S Company, Berlin, Germany) according to the procedures reported by Feng et al. with some modifications [33], which was equipped with an autosampling unit using an automatic headspace injection. The capillary column was an MXT-5 (15 m × 0.53 mm ID). For the analysis, 1 mL of sea buckthorn juice was placed in a 20 mL headspace vial and incubated at a temperature of 50 °C for 20 min. The injection needle temperature was set at 85.0 °C, and the column temperature was maintained at 60 °C. Nitrogen, with a purity of 99.99%, served as the carrier gas.

### 2.11. Statistical Analysis

All experiments were carried out in triplicate, and the data were presented as the mean ± standard deviation. Multigroup analyses were performed using one-way analysis of variance (ANOVA) using SPSS software 29.0 (SPSS Inc., Chicago, IL, USA). A *p*-value of < 0.05 indicated a statistically significant difference. Origin 2021 (Micro Software, Inc., Pittsfield, MA, USA) software was used to do principal component analysis (PCA) and peak area statistics using GC-IMS VOCs. GraphPad Prism 8 (GraphPad Company, San Diego, CA, USA) was used for the drawings. Online software was used to generate the stacked column chart of aroma components (https://chiplot.com, accessed on 12 October 2022).

## 3. Results and Discussion

### 3.1. Effects of TP and PEF-HPP on the Aerobic Plate Count of Sea Buckthorn

The microbiological quality outcomes after sterilization of sea buckthorn juice are displayed in Table 1. It was evident that the aerobic plate count of the CK was 2.2 × 10^3^ CFU/mL. Following PEF-HPP and TP processing, the aerobic plate count was not detectable in the sea buckthorn juice samples. The findings showed that both processing techniques effectively eradicated germs from the juice and guaranteed food safety. These findings are consistent with a study by Gan et al., who reported that bacteria could be effectively removed from pear juice using both heat treatment settings and HPP (500 MPa, 20 min) [34].

### 3.2. Effects of TP and PEF-HPP on the Juice Yield of Sea Buckthorn

**Figure 2:**
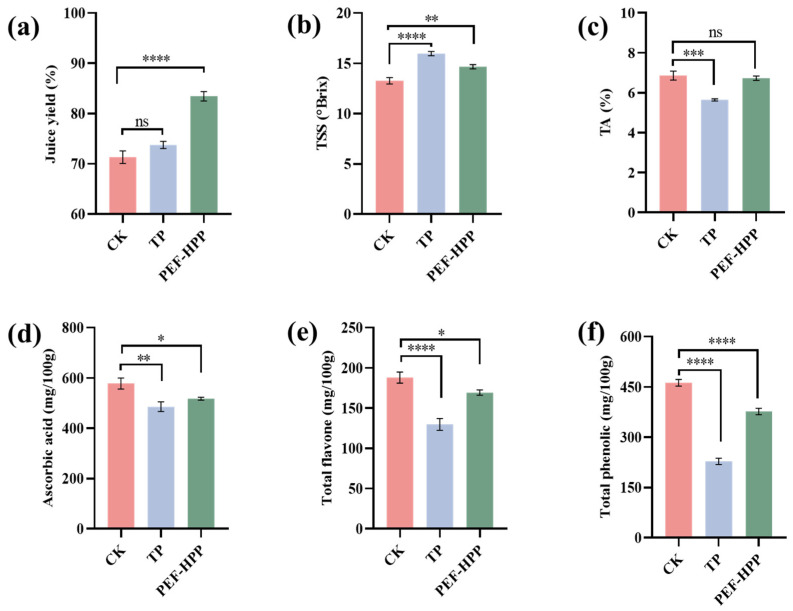
Composition characteristics of different treatment juices: (**a**) juice yield; (**b**) TSS; (**c**) TA; (**d**) ascorbic acid content; (**e**) total flavone content; (**f**) total phenol content. Asterisks indicate statistical significance at different levels of adjusted *p*-values: ns, *p* > 0.05; * *p* < 0.05; ** *p* < 0.01; *** *p* < 0.005; **** *p* < 0.001.

The effectiveness of the two procedures in determining the juice yield was assessed by weighing the sea buckthorn juice collected following TP and PEF-HPP treatments. The juice yield of sea buckthorn was 72.1% after TP treatment and 83.47% after PEF-HPP treatment (Figure 2a). This resulted in a 12% increase in sea buckthorn juice yield (71.34% in the CK). The PEF acted on the sea buckthorn pulp cells, exerting an electroporation effect on the cell membrane, and the cell content flowed out, effectively improving the juice yield [35]. It was discovered that PEF treatment of blueberry juice causes the cell wall to be pitted and folded, as well as severe damage to the cell membrane, with obvious holes appearing, causing the cell contents to leak out, which is an important reason for increasing juice yield [36].

### 3.3. Effects of TP and PEF-HPP on Nutritional Compounds

As shown in Figure 2b, the TSS content of TP and PEF-HPP processed sea buckthorn juice was significantly increased. Xu et al., found that the phenomenon is mostly caused by the degradation of soluble components like pectin in fruit juices [37]. TA content in sea buckthorn juice tended to decrease significantly (*p* < 0.005) after TP treatment, which may be caused by the instantaneous high temperature leading to the Maillard reaction and degradation and volatilization of low-boiling acid, thus reducing the TA content [21].

Ascorbic acid has an unstable structure, which is susceptible to changes in temperature, humidity, pressure, friction, light, and acid. As shown in Figure 2d, TP treatment significantly decreased ascorbic acid content (*p* < 0.01). HPP accelerates the metabolic processes in food items. It also speeds up the oxidation of ascorbic acid when reactive oxygen species come in contact with it [7]. Naliyadhara et al. found that PEF has a negligible negative impact on ascorbic acid [38]. Polyphenols, including phenols and flavonoids, are plant secondary-level metabolites with free radical scavenging and antioxidant functions; they are the source of many of the flavor chemicals found in apple citrus fruits [39]. The influence of different processing methods on total flavone and total phenolic of sea buckthorn juice is shown in Figure 2e,f. Total flavonoids in sea buckthorn juice were severely lost to 129.8 mg/100 g after TP treatment (180.03 mg/100 g in CK). A similar phenomenon was found in total flavonoid concentrations of durian puree after TP and HPP processing; TP retained only 80% of the flavonoids [40]. The decrease in content was attributed to structural damage, with high temperatures causing the degradation of flavonoids [41]. The polyphenol retention rate in sea buckthorn juice after processing was 49.32% (TP) and 81.48% (PEF-HPP). Although the total phenol content of sea buckthorn juice after PEF-HPP processing was significantly reduced (*p* < 0.001), the oxidation and degradation of phenolics were slowed down as the processing was carried out at ambient temperature and under airtight conditions [42]. This is consistent with the results of studies on carrot puree [43]. Li et al. found that PEH-HPP could not completely deactivate POD and PPO enzymes, and the remaining enzymes oxidized the beneficial components [44].

### 3.4. Effects of TP and PEF-HPP on Organic Acid Content

**Figure 3:**
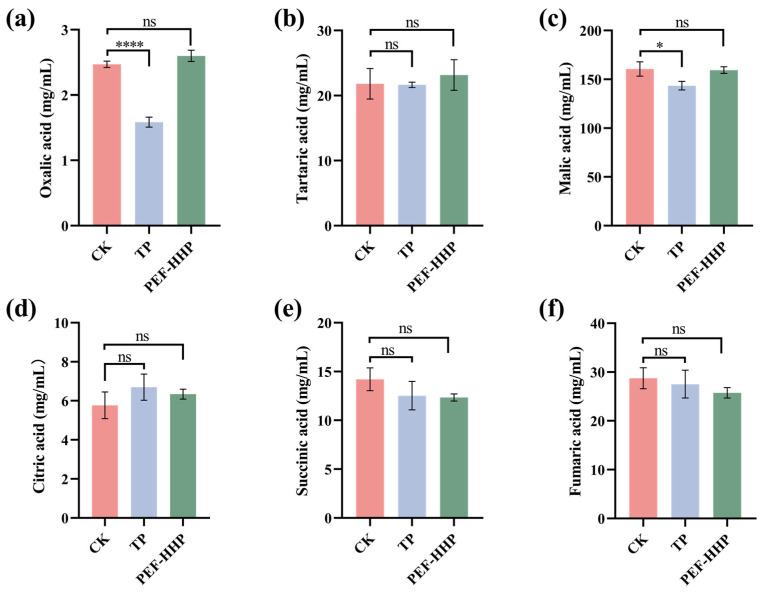
Organic acid content of different treatment juices: (**a**) oxalic acid; (**b**) tartaric acid; (**c**) malic acid; (**d**) citric acid; (**e**) succinic acid; (**f**) fumaric acid. Asterisks indicate statistical significance at different levels of adjusted *p*-values: ns, *p* > 0.05; * *p* < 0.05; **** *p* < 0.001.

Sea buckthorn is rich in organic acids, more commonly malic acid, citric acid, and tartaric acid, and their content and distribution affect the organoleptic quality of sea buckthorn juice, contributing to its distinctive acidic taste [45]. The content of organic acids in sea buckthorn juice is shown in Figure 3 and Figure S1. The analysis revealed that the malic acid content in fresh sea buckthorn juice was approximately 160.59 mg/mL, constituting roughly 59.60% of the organic acid content. PEF-HPP treatment did not affect the content of all six organic acids, suggesting that the treatment did not change the taste of sea buckthorn juice. These findings aligned with the results reported by Wibowo et al. [46]. However, the contents of malic acid and oxalic acid in sea buckthorn juice were significantly reduced in TP (*p* < 0.05). The study by Niu et al. [47] observed a 20.5% reduction in malic acid content and a decrease in oxalic acid content in TP. This reduction was attributed to the acceleration of the degradation of malic acid, oxalic acid, and similar compounds at elevated temperatures, which promoted metabolic reactions.

### 3.5. Effects of TP and PEF-HPP on Color Characteristics

Color is an important benchmark for evaluating the quality of fruit and vegetable juices, and various natural pigments such as anthocyanins and carotenoids brown fruit and vegetable juices during processing, affecting their color [48]. According to Table 1, following TP and PEF-HPP processing, the C^*^ of sea buckthorn juice fell, as did the color saturation. There was no significant difference (*p* > 0.05) in the *h*^0^ values between the three groups of sea buckthorn juice. Sea buckthorn juice maintained good color characteristics after PEF-HPP treatment, while TP treatment caused significant changes in Δ*E*. The results showed that the ΔE of sea buckthorn juice after PEF-HPP treatment was 2.67, with a slightly obvious color change (1.5 < Δ*E* < 3.0, slightly visible), while the Δ*E* of sea buckthorn juice after TP treatment was 4.49, with a completely obvious color change (3.0 < Δ*E* < 6.0, completely visible) and the browning degree increased significantly. This phenomenon is mostly caused by the lowering of a^*^ and b^*^ after TP treatment, which could be attributed to the oxidative degradation of anthocyanins and carotenoids in sea buckthorn as well as the Maillard reaction. HPP and PEF processing, on the other hand, can minimize degradation and enzymatic browning while causing minimal color change because they are performed at ambient temperature and under airtight conditions. Xu et al. and Huang et al. reported similar results when processing persimmon and carrot juices at high pressure [16,17].

### 3.6. Effects of TP and PEF-HPP on Particle Size Distribution and Viscosity

**Figure 4:**
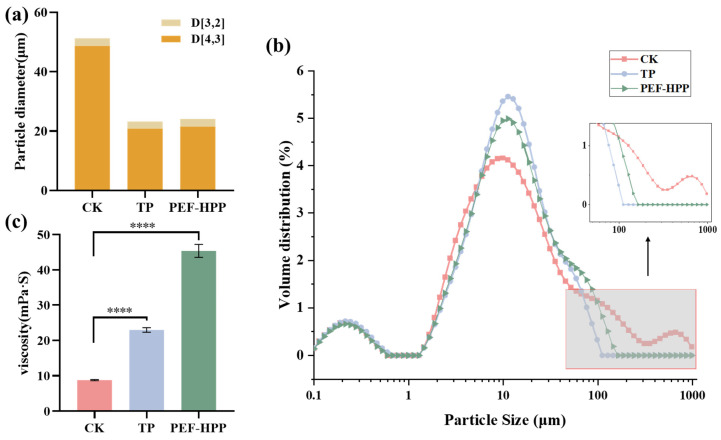
Particle size and viscosity of different treatment juices: (**a**) average particle size: D[4,3] (volume-weighted mean diameter usually affected by bigger particles) and D[3,2] (surface-weighted mean diameter influenced by smaller particles); (**b**) particle size distribution; (**c**) viscosity. Asterisks indicate statistical significance at different levels of adjusted *p*-values: **** *p* < 0.001.

Some particles in CK exceeded 1000 μm, and both TP and PEF-HPP treatments could reduce the particle size of sea buckthorn juice (Figure 4a,b). Higher D[3,2] values were associated with an increased likelihood of aggregate formation between particles, whereas elevated D[4,3] values suggested the presence of heavier particles in the juice system, making them more prone to precipitation [27]. In a cloudy hawthorn juice system, a similar trend was also observed [37]. Processing reduced D[4,3], with PEF and HPP causing cell lysis, TP dissolving pectin, and partial cell wall detachment contributing to the reduction in particle size in the juice [49].

As shown in Figure 4c, the viscosity of sea buckthorn juice in the CK was 8.77 mPa·S; the viscosity of sea buckthorn juice significantly increased after TP and PEF-HPP processing (*p* < 0.001). The decrease in particle size resulted in an increase in interfacial area and a decrease in the average distance between particles, leading to stronger interparticle interactions and an increase in juice viscosity [50]. The increase in viscosity in fruit juices is also linked to the inactivation of endogenous enzymes found in fruits and vegetables, such as POD and PPO [51]. The investigation of processed spinach and mango juices also revealed an increase in viscosity, indicating that PEF-HPP and TP treatments increased pectin release, contributing to an increase in juice viscosity and consequently reducing the settling of suspended matter in the sea buckthorn juice system [28,52].

### 3.7. Effects of TP and PEF-HPP on Enzyme Activities

**Figure 5:**
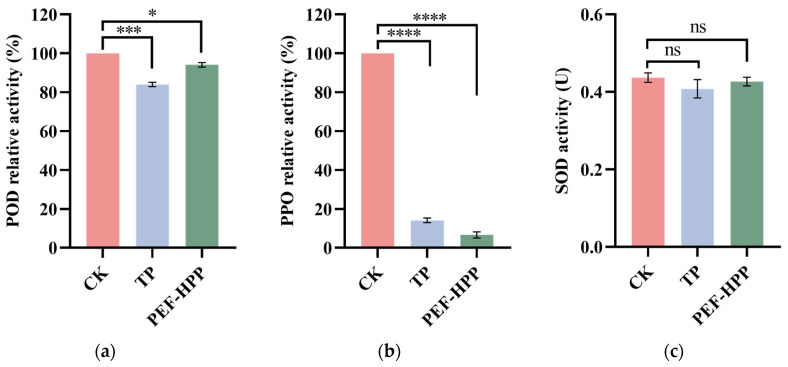
POD relative activity (**a**), PPO relative activity (**b**), and SOD activity (**c**) in different treatment juices. Asterisks indicate statistical significance at different levels of adjusted *p*-values: ns, *p*-value > 0.05; * *p* < 0.05; *** *p* < 0.005; **** *p* < 0.001.

The activities of POD and PPO were directly related to the quality of sea buckthorn juice. POD and PPO participated in juice browning by catalyzing phenolic oxidation in chloroplasts, and reducing PPO activity had beneficial effects on the color and flavor of the juice [53]. PEF-HPP caused a decrease in POD and PPO activity (Figure 5a,b). The inhibitory effect of HPP and PEF on PPO was more pronounced, favoring a reduction in the browning degree of sea buckthorn juice. High pressure acted as an inhibitor of enzyme activity by inducing changes in the molar volume of the enzyme through compaction, leading to changes in its conception and subsequent loss of enzyme function [54]. Huang et al. found that POD and PPO exhibited different sensitivities to high voltage and electric fields due to various factors such as enzyme source, temperature, and system [55].

As shown in Figure 5c, PEF-HPP and TP had no significant effect on the SOD activity of sea buckthorn juice (*p* > 0.05). SOD is highly resistant to high temperatures, and TP could not significantly reduce its activity [56]. Liu et al., reported the SOD activity of both fresh and HPP juice was 470.74 U/g FW, indicating that SOD was insensitive to high pressure and could maintain good stability under each condition [57].

### 3.8. Effects of TP and PEF-HPP on Flavor Compounds

#### 3.8.1. Analysis of VOCs in Sea Buckthorn Juice Using an E-Nose

**Figure 6:**
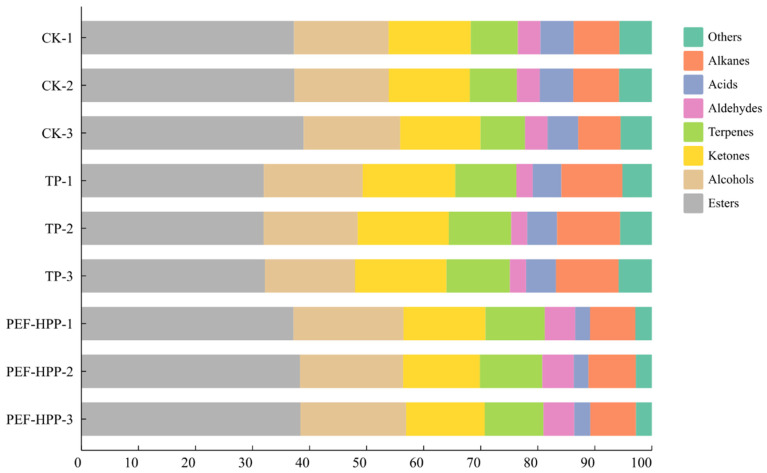
Classification of VOCs detected in different treatment juices by an E-nose.

Fruit aroma is predominantly contributed by various volatile esters present in fruits, which are often fruity and floral, imparting a fresh, sweet flavor and mouthfeel to sea buckthorn juice [58]. As shown in Figure 6, the ester level of TP-processed sea buckthorn juice (32.00%) fell significantly (*p* < 0.001), but the PEF-HPP group (37.93%) did not differ significantly from fresh sea buckthorn juice (37.86%). This is congruent with the findings of Wu et al., who processed pineapple juice and discovered that PEF and HPP had less of an influence on the juice’s esters, allowing for improved retention [7]. Alcohols constitute the second-largest group of volatiles in sea buckthorn juice. The alcohol content in sea buckthorn juice increased after PEF-HPP processing (18.62%). PEF and HPP were discovered to potentially produce additional aromatic chemicals while processing orange juice [59]. The highest content of ketones was observed after TP. Ketones are mainly produced by the oxidation of unsaturated fatty acids or by microbial metabolism during fermentation. Heating promoted oxidation, reduction, and hydrolysis reactions in sea buckthorn juice, resulting in the breakdown and transformation of alcohols, ketones, and ester [60].

#### 3.8.2. Qualitative Analysis of VOCs in Sea Buckthorn Juice Using GC-IMS

##### HS-GC–IMS Topography of VOCs in Sea Buckthorn Juice

**Figure 7:**
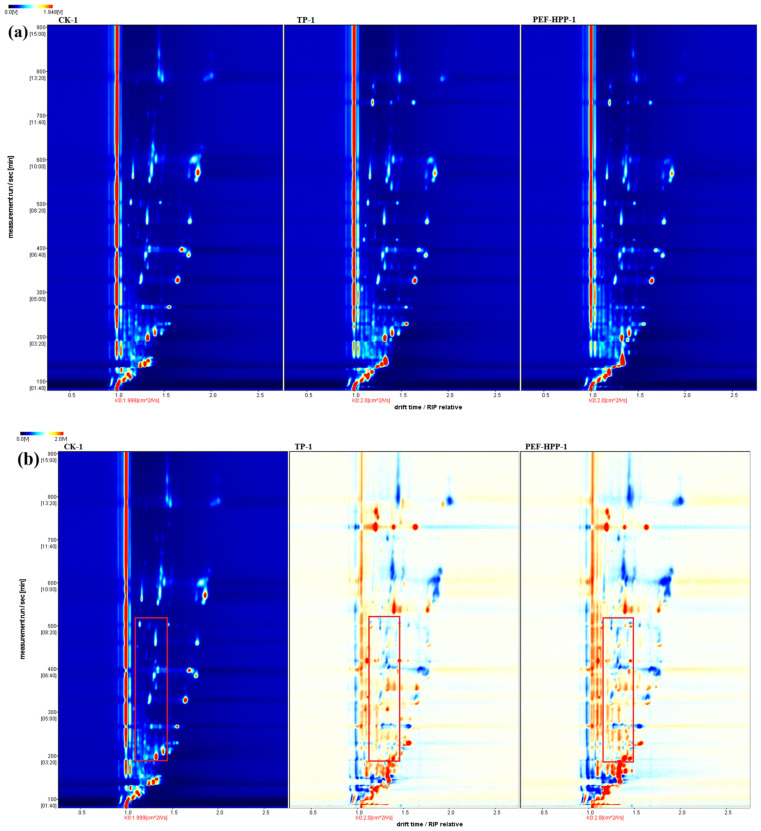
Topographic plots (**a**) and topographic sub-traction plots (**b**) in different treatments of sea buckthorn juice.

The topographic plots of VOCs in sea buckthorn juice from the three different processings are shown in Figure 7a. In these topographic plots, the vertical coordinate signifies the retention time of the gas phase, the horizontal coordinate represents the drift time of ion migration, and the red vertical line on the left denotes the reactive ion peak (RIP). Each dot to the right side of the RIP represents a volatile compound, with the color of the dot indicating the concentration of this substance: white indicating low concentration and red indicating high concentration. Most signals in the graph appear at retention times of 100–900 s and drift times of 1.0–2.0 s. A difference comparison model was used to address differences between the three sample groups by comparing sea buckthorn juice samples subjected to various treatments (Figure 7b).

**Figure 8:**
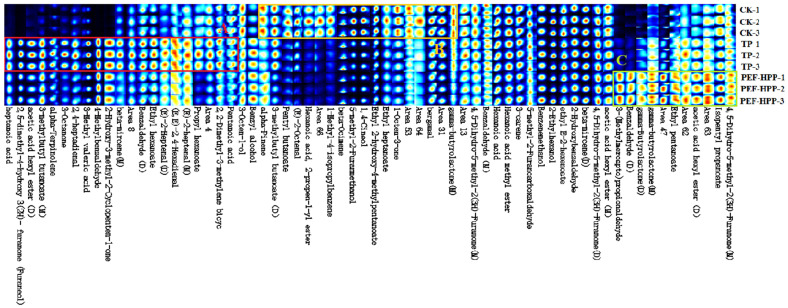
Fingerprints of VOCs detected in different treatment juices by GC-IMS.

The CK served as the reference, and the spectra of the other two groups of samples were subtracted from the reference. In the subtracted spectra, white indicated the cancellation of the same substance concentration, red indicated a higher concentration than in the reference, and blue indicated a lower concentration than in the reference, with darker colors indicating significant differences. In drift times of 1.0–1.5 s, the concentration of VOCs in sea buckthorn juice after PEF-HPP was significantly higher than TP and CK. The concentrations of VOCs in the juice after PEF-HPP and TP at 1.5–2.0 s were lower than those in the CK. To precisely determine the specific composition of different compounds, researchers required in-depth statistical analyses of the combined volatility profile information from different samples (Table 2 and Figure 8).

##### Qualitative Analysis of VOCs under Different Treatments of Sea Buckthorn Juice

The fingerprints of all the analyzed peaks are shown in Figure 8. The color of each peak signifies VOC content, with darker colors indicating higher concentrations. These fingerprints facilitated the comparison of dynamic changes in VOCs across different samples. As shown in Figure 8 and Table 2, in Region A, the TP had the highest content of VOCs in sea buckthorn juice. However, heptanoic acid, pentanoic acid, alpha-terpinolene, 2,4-heptadienal, and 3-methyl valeric acid led to a stronger, deteriorated sharp sourness and a pungent acidic flavor in TP. Yi et al. discovered that the synthesis of these compounds could be due to degradation with unsaturated fatty acids, hydrolysis, and oxidative reactions with phenols, ketones, and aldehydes [61]. Region B showcased exclusive VOCs in the CK. In HPP and PEF circumstances, various enzymes associated with lipid metabolism may be triggered, leading to a reduction of acids and aldehydes in the aroma [62]. It has been shown that alpha-pinene, the most important terpene in citrus fruits, is lost during processing, but this loss is more pronounced with TP. High temperatures cause enzyme inactivation, which degrades ascorbic acid, sugars, amino acids, and unsaturated fatty acids in juice, affecting VOC release [63]. Region C showed that the PEF-HPP had the highest VOC content, gamma-butyrolactone, ethyl pentanoate, and acetic acid hexyl ester, all of which provided sweet, plum and apple flavors to the sea buckthorn juice. Further, all these VOCs were among the esters with the highest content of the sea buckthorn representative aroma, such as isopentyl propanoate, acetic acid hexyl ester, and ethyl pentanoate [64]. A series of complex reactions during PEF-HPP treatment may be attributed to the emergence of new substances [65].

##### PCA of VOCs in Different Treatments of Sea Buckthorn Juice

**Figure 9:**
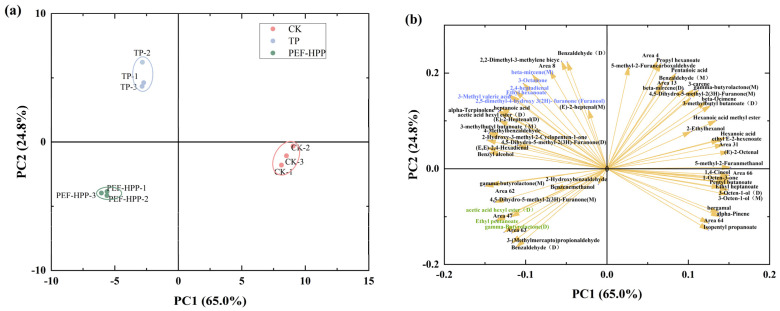
Principal component analysis of VOCs in different treatment juices: (**a**) score plot and (**b**) loading plot.

Principal component analysis (PCA) was performed based on the peak areas of VOCs (Figure 9). PCA has a distinctive advantage in classifying samples based on multiple data variables [33]. The combined contribution of PC1 and PC2 accounted for 89.8%, which was sufficient to explain the similarities between the different treatments (Figure 9a). PC1 effectively distinguished unprocessed sea buckthorn juice from processed versions, and PC2 discriminated CK and PEF-HPP from TP. Although PEF-HPP treatment did not completely preserve the fresh flavor, it offers a good alternative to TP.

To display the correlation between VOCs and samples, we show the loading plot (Figure 9b). Some of the areas in Figure are dispersed, showing that there are disparities across the sample groups due to the presence of certain VOCs. The TP samples are primarily dispersed in the IV quadrant and are distinguished by the presence of characteristic scents such as 3-octanone, 2,4-heptadienal, 3-methyl valeric acid, ethyl hexanoate, 2,5-Dimethyl-4-hydroxy-3(2H)-furanone (furaneol), and beta-mircene (M). The PEF-HPP samples were primarily distributed in the III quadrant, with ethyl-pentanoate, gamma-butyrolactone (D), and acetic acid hexyl ester (D) as prominent scents. The characteristic fragrance was ester (D). The PCA results were consistent with Figure 8, which better demonstrated the changes in characteristic flavor compounds following various processing procedures. Overall, GC-IMS is a quick analytical method for distinguishing between the flavors of different processed sea buckthorn liquids and detecting changes in signature flavor components.

## 4. Conclusions

The present investigation aimed to evaluate the effects of three different treatments (CK, TP, and PEF-HPP) of sea buckthorn juice. This work found that the synergistic treatment of PEF (5000 V, 30 μs) and HPP (500 MPa, 15 min) ensured the food safety of sea buckthorn juice and boosted yield by 11.37% compared to TP. Furthermore, PEF-HPP enhanced the nutritional retention of sea buckthorn juice by 90% on average, while inhibiting enzyme activity improved its color, viscosity, and particle size. The flavors of different processing sea buckthorn juices could be well distinguished using an E-nose and GC-IMS. PEF-HPP prevents the aldehydes and acids produced by high temperatures from harming the VOCs, and it can improve the aroma of sea buckthorn juice. PEF-HPP technology has the potential to create a new and effective non-thermal processing method for sea buckthorn juice, as well as increase its processing efficiency. However, changes in nutrient content by PEF-HPP and TP processing were related to component interactions between nutrients. Therefore, future work still needs to further investigate the phenomenon of component interactions during processing.

## Figures and Tables

**Table 1:** Aerobic plate count and color characteristics of different treatment juices.

Sample	Aerobic Plate Count (CFU/mL)	Color Characteristics					
		L*	a*	b*	Δ*E*	C*	*h* ^0^
CK	2.2 × 10^3^ ± 3.43	54.79 ± 0.04 ^a^	39.19 ± 0.51 ^a^	49.94 ± 1.16 ^a^	Control	63.49 ± 1.06 ^a^	0.91 ± 0.01 ^a^
TP	ND	53.76 ± 0.68 ^a^	36.58 ± 0.29 ^c^	46.43 ± 1.28 ^b^	4.49 ± 1.28 ^b^	59.12 ± 1.19 ^b^	0.90 ± 0.01 ^a^
PEF-HHP	ND	54.89 ± 0.59 ^a^	37.72 ± 0.24 ^b^	47.71 ± 0.40 ^ab^	2.67 ± 1.04 ^a^	60.82 ± 0.30 ^b^	0.90 ± 0.01 ^a^

Note: ND, not detectable (<10 CFU/mL). Different superscripts ^(a–c)^ indicate significant differences (*p* < 0.05) within a specific row.

**Table 2:** Identified VOCs of three treatment sea buckthorn juices based on GC-IMS.

	Compound	CAS#	Formula	MW	RI	Rt [s]	Dt [a,u]	Odor Series	Odor Description
Esters	Propyl hexanoate	C626777	C_9_H_18_O_2_	158.2	1089	784.08	1.94	Fruity	Sweet, fruity, juicy, pineapple, green, tropical *
	Pentyl butanoate	C540181	C9H_18_O_2_	158.2	1090	791.13	1.45	Fruity	Sweet, fruity, banana, pineapple, cherry, tropical ^#^
	3-Methylbutyl butanoate (D)	C106274	C_9_H_18_O_2_	158.2	1059.7	602.55	1.42	Fruity	Fruity, green, apricot, pear, banana *
	Acetic acid hexyl ester (D)	C142927	C_8_H_16_O_2_	144.2	1019.7	420.26	1.46	Fruity	Fruity, green, apple, banana, sweet ^#^
	Ethyl hexanoate	C123660	C_8_H_16_O_2_	144.2	1002	358.46	1.33	Fruity	Sweet, fruity, pineapple, waxy, green, banana *
	Ethyl 2-hydroxy-4-methylpentanoate	C10348477	C_8_H_16_O_3_	160.2	1053.8	571.15	1.86	Fruity	Fresh, blackberry ^#^
	4,5-Dihydro-5-methyl-2(3H)-furanone (D)	C108292	C_5_H_8_O_2_	100.1	939	213.50	1.40	Herbal	Herbal, sweet, tobacco, cocoa ^#^
	Acetic acid hexyl ester (D)	C142927	C_8_H_16_O_2_	144.2	1013.5	397.49	1.44	Fruity	Fruity, green, apple, banana, sweet ^#^
	Isopentyl propanoate	C105680	C_8_H_16_O_2_	144.2	966.6	267.37	1.37	Fruity	Sweet, fruity, banana, pineapple ^#^
	gamma-Butyrolactone (M)	C96480	C_4_H_6_O_2_	86.1	903.3	159.74	1.10	Creamy	Creamy, oily, fatty, caramellic ^#^
	4,5-Dihydro-5-methyl-2(3H)-furanone (M)	C108292	C_5_H_8_O_2_	100.1	944.1	222.58	1.09	Herbal	Herbal, sweet, tobacco, cocoa ^#^
	Ethyl heptanoate	C106309	C_9_H_18_O_2_	158.2	1096.9	841.53	1.44	Fruity	Fruity, pineapple, cognac, rummy, winey *
	Hexanoic acid, 2-propen-1-yl ester	C123682	C_9_H_16_O_2_	156.2	1077	703.71	1.33	Fruity	Sweet, fruity, pineapple, tropical ^#^
	Acetic acid hexyl ester (M)	C142927	C_8_H_16_O_2_	144.2	1013.3	396.61	1.35	Fruity	Fruity, green, apple, banana, sweet ^#^
	Ethyl pentanoate	C539822	C_7_H_14_O_2_	130.2	903.4	159.85	1.27	Fruity	Sweet, fruity, apple, pineapple, green, tropical *
	gamma-Butyrolactone (M)	C96480	C_4_H_6_O_2_	86.1	915.8	176.75	1.05	Creamy	Creamy, oily, fatty, caramellic ^#^
	Hexanoic acid methyl ester	C106707	C_7_H_14_O_2_	130.2	930.5	199.37	1.33	Pome	Fruit, fresh, sweet *
	gamma-Butyrolactone (D)	C96480	C_4_H_6_O_2_	86.1	921.2	184.70	1.34	Creamy	Creamy, oily, fatty, caramellic ^#^
	4,5-Dihydro-5-methyl-2(3H)-furanone (M)	C108292	C_5_H_8_O_2_	100.1	957.9	249.04	1.09	Herbal	Herbal, sweet, tobacco, cocoa ^#^
	3-Methylbutyl butanoate (M)	C106274	C_9_H_18_O_2_	158.2	1047.4	539.28	1.40	Fruity	Fruity, green, apricot, pear, banana *
	ethyl E-2-hexenoate	C27829727	C_8_H_14_O_2_	142.2	1030.4	462.58	1.32	Fruity	Fruity, green, sweet, juicy ^#^
Alcohols	3-Octen-1-ol (D)	C20125842	C_8_H_16_O	128.2	1061.4	611.84	1.38	Fatty	Fresh, fatty, greasy, melon, green ^#^
	3-Octen-1-ol (M)	C20125842	C_8_H_16_O	128.2	1055.1	578.17	1.37	Fatty	Fresh, fatty, greasy, melon, green ^#^
	2-Ethylhexanol	C104767	C_8_H_18_O	130.2	1030.1	461.65	1.78	Floral	Rose, green *
	5-Methyl-2-furanmethanol	C3857258	C_6_H_8_O_2_	112.1	967	268.25	1.56	Fatty	Fatty, greasy, green ^#^
	Benzyl alcohol	C100516	C_7_H_8_O	108.1	1027.9	452.57	1.13	Floral	Floral, rose, phenolic, balsamic *
	Benzenemethanol	C100516	C_7_H_8_O	108.1	1039.4	501.63	1.46	Floral	Floral, rose, phenolic, balsamic *
Aldehydes	4-Methylbenzaldehyde	C104870	C_8_H_8_O	120.2	1080.9	729.06	1.21	Fruity	Fruity, cherry, phenolic ^#^
	(*E*)-2-Octenal	C2548870	C_8_H_14_O	126.2	1059.2	599.68	1.82	Herbs	Green, nut, fat *
	Benzaldehyde (D)	C100527	C_7_H_6_O	106.1	936.3	208.86	1.47	Maillard	Almond, burned sugar *
	Benzaldehyde (M)	C100527	C_7_H_6_O	106.1	945.2	224.62	1.13	Maillard	Almond, burned sugar *
	Bergamal	C106729	C_9_H_16_O	140.2	1050.5	554.52	1.35	Vegetable	Fruit, green, melon *
	2,4-Heptadienal	C5910850	C_7_H_10_O	110.2	1001.4	356.47	1.65	Green	Green, pungent, spicy ^#^
	Benzaldehyde (D)	C100527	C_7_H_6_O	106.1	926.6	193.11	1.46	Maillard	Almond, burned sugar *
	(*E*)-2-Heptenal (D)	C18829555	C_7_H_12_O	112.2	967.7	269.70	1.21	Edible Oil	Soap, fat *
	5-Methyl-2-furancarboxaldehyde	C620020	C_6_H_6_O_2_	110.1	956.5	246.27	1.14	Maillard	Almond, caramel, burned sugar *
	(*E*)-2-Heptenal (M)	C18829555	C_7_H_12_O	112.2	957.2	247.78	1.21	Edible Oil	Soap, fat *
	(*E*,*E*)-2,4-Hexadienal	C142836	C_6_H_8_O	96.1	919.5	182.19	1.14	Green	Green, spicy, citrus ^#^
	2-Hydroxybenzaldehyde	C90028	C_7_H_6_O_2_	122.1	1039.3	501.13	1.15	Spicy	Spicy, medicinal, astringent ^#^
	3-(Methylmercapto)propionaldehyde	C3268493	C_4_H_8_OS	104.2	907.2	164.89	1.41	Vegetable	Potato, tomato, vegetable, creamy ^#^
Ketones	3-Octanone	C106683	C_8_H_16_O	128.2	983.6	307.13	1.33	Herbs	Herb, butter, resin *
	2-Hydroxy-3-methyl-2-cyclopenten-1-one	C80717	C_6_H_8_O_2_	112.1	1019.2	418.48	1.11	Caramellic	Caramellic, maple ^#^
	2,5-Dimethyl-4-hydroxy-3(2H)-furanone (Furaneol)	C3658773	C_6_H_8_O_3_	128.1	1081.1	730.36	1.63	Caramellic	Sweet, candy, strawberry, cotton, sugar ^#^
	1-Octen-3-one	C4312996	C_8_H_14_O	126.2	966.8	267.78	1.28	Earthy	Herbal, mushroom, earthy, musty ^#^
Terpenes	3-Carene	C13466789	C_10_H_16_	136.2	1010.1	385.40	1.30	Citrus	Lemon, resin *
	beta-Mircene (D)	C123353	C_10_H_16_	136.2	991.5	327.37	1.65	Fermented	Balsamic, must, spice *
	2,2-Dimethyl-3-methylene-bicyc	C79925	C_10_H_16_	136.2	949.1	231.87	1.21	Woody	Woody, herbal ^#^
	alpha-Terpinolene	C586629	C_10_H_16_	136.2	1086.6	767.21	1.21	Aromatic	Pine, plastic *
	beta-Ocimene	C13877913	C_10_H_16_	136.2	1052.6	565.12	1.17	Citrus	Sweet, herb *
	beta-Mircene (M)	C123353	C_10_H_16_	136.2	983.9	307.90	1.09	Fermented	Balsamic, must, spice *
	alpha-Pinene	C80568	C_10_H_16_	136.2	941	217.04	1.25	Herbal	Fresh, sweet, pine ^#^
	1,4-Cineole	C470677	C_10_H_18_O	154.3	1013	395.74	1.69	Herbal	Pine, minty, green ^#^
Acids	Heptanoic acid	C111148	C_7_H_14_O_2_	130.2	1081.1	730.01	1.40	Cheesy	Rancid, sour, cheesy ^#^
	Hexanoic acid	C142621	C_6_H_12_O_2_	116.2	992	328.70	1.26	Fatty	Sour, fatty, sweat *
	3-Methyl valeric acid	C105431	C_6_H_12_O_2_	116.2	947.6	229.14	1.56	Animal	Sharp, acidic, green ^#^
	Pentanoic acid	C109524	C_5_H_10_O_2_	102.1	929.5	197.64	1.23	Cheesy	Acidic, sharp, sweaty, fruity ^#^

Note: D, dimer; Dt, drift time; MW, molecular weight; M, monomer; RI, reserved index; Rt, retention time. * Odor descriptions are from the website http://www.flavornet.org/flavornet.html (accessed on 25 September 2022). ^#^ Odor descriptions are from the website http://www.thegoodscentscompany.com/ (accessed on 25 September 2022).

## Data Availability

The original contributions presented in the study are included in the article, further inquiries can be directed to the corresponding author.

## Supplementary Materials

The following supporting information can be downloaded at: https://www.mdpi.com/article/10.3390/foods13121829/s1, Figure S1: Organic acid content HPLC chromatogram of standard (a) and sea buckthorn juice (b).

## Abbreviations

TP	thermal processing
PEF	pulsed electric field
HPP	high-pressure processing
VOCs	volatile organic compounds
HS-GC–IMS	headspace chromatography–ion mobility spectrometer
CK	control group
POD	peroxidase
PPO	polyphenol oxidase
TSS	total soluble solid
TA	titratable acidity
HPLC	high-performance liquid chromatography
PDA	photodiode array detector
PCA	principal component analysis
SOD	superoxide dismutase
RIP	reactive ion peak
