# Supplementary material for: Effects of Pulsed Electric Field and High-Pressure Processing Treatments on the Juice Yield and Quality of Sea Buckthorn
Source: Foods. 2024 Jun 11;13(12):1829. doi: 10.3390/foods13121829 (PMC11202788; doi:10.3390/foods13121829)
Supplement: Supplementary file 1 [file foods-13-01829-s001.zip › foods-2998176-supplementary.pdf]

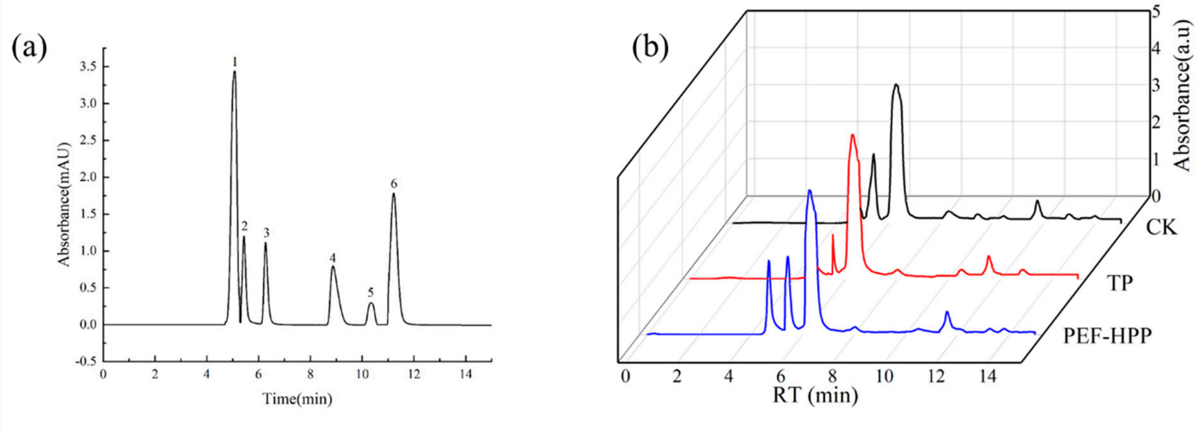

Supplemental Figure S1. Organic acid content HPLC chromatogram of standard (a) and sea buckthorn juice (b)

Note: (a)&(b): 1: oxalic acid; 2: tartaric acid; 3: malic acid; 4: citric acid; 5: succinic acid; 6: fumaric acid.
